# Efficacy of the Measures Adopted to Prevent COVID-19 Outbreaks in an Italian Correctional Facility for Inmates Affected by Chronic Diseases

**DOI:** 10.3389/fpubh.2021.694795

**Published:** 2021-07-07

**Authors:** Angela Stufano, Nicola Buonvino, Francesco Cagnazzo, Nicola Armenise, Daniela Pontrelli, Giovanna Curzio, Leonarda De Benedictis, Piero Lovreglio

**Affiliations:** ^1^Interdisciplinary Department of Medicine - Section of Occupational Medicine, University of Bari, Bari, Italy; ^2^U.O.C. Penitentiary Medicine – Department of Territorial Care, Bari Local Health Authority, Bari, Italy

**Keywords:** SARS-CoV-2 infection, congregate settings, prison, COVID - 19 outbreak, antigen test

## Abstract

**Background:** COVID-19 outbreaks in prisons and jails may affect both inmates and correctional workers. An observational study has been performed to investigate the efficacy of specific procedures and of a serial testing approach adopted for the COVID-19 prevention in an Italian correctional facility (Bari, Apulia) for inmates affected by chronic diseases.

**Methods:** Two SARS-CoV-2 antigen testing campaigns were carried out for all the prisoners and correctional workers, including correctional officers (CO), administrative staff (AS), correctional health care workers (HCW), and operators working with people completing their sentence outside the prison (OOP). Antigen testing was conducted on nasopharyngeal swab specimens, using a fluorescence immunoassay for the qualitative detection of nucleocapsid SARS-CoV-2 antigen. All subjects positive to the antigen test underwent confirmation by rRT-PCR test.

**Results:** In total, 426 new and residential inmates were tested during the first campaign and 480 during the second campaign. Only two new inmates resulted positive at the first campaign, while no positive cases were observed at the second campaign or outside of the testing campaigns. In total, 367 correctional workers were tested at the first campaign and 325 at the second. At the first, 4 CO and 2 HCW showed positive test results, while no new positive cases were observed at the second. Moreover, 1 CO and 1 HCW resulted positive outside of the testing campaigns for the onset of symptoms while at home.

**Conclusion:** The implementation of a full risk management plan in a correctional facility, including both a strict protocol for the application of preventive measures and a serial testing approach, seems to be able to prevent COVID-19 outbreaks in both inmates and correctional workers.

## Background

Transmission of Severe Acute Respiratory Syndrome—Coronavirus 2 (SARS-CoV-2) by asymptomatic, pre-symptomatic, and mildly symptomatic infected subjects poses the challenge of how to control the COVID-19 spread in correctional and detention facilities (1). In fact, unavoidable close contact, and sharing often overcrowded and poorly ventilated confined spaces, can easily favor virus infection both by droplets and airborne transmission (2). Moreover, many inmates may be highly vulnerable to severe COVID-19, due to the generally poor health profiles and more limited access to health care services (3). The management of the COVID-19 in prisons and jails, therefore, needs also to be considered according to the debated implications that the pandemic situation may have in these settings, such as the possible incompatibility with the prison regime of the vulnerable inmates leading to their accelerated release, the access to the care for detained COVID-19 patients, the legal obligation of protecting inmates from COVID-19 outbreaks inside the facilities.

Higher COVID-19 incidence rates have been reported in many correction and detention facilities worldwide: the UK, Italy, US, Iran, and Latin America countries reported outbreaks in the majority of the prisons, particularly if operating at far beyond their capacity (4–6). However, preventing the transmission of COVID-19 within correctional and detention facilities should be an integral part of the public health response to the current pandemic, also because COVID-19 outbreaks may affect both inmates and correctional workers (7, 8). Particularly, the protection of the latter should be a key point of the prevention measures, in view of their daily interaction with both incarcerated persons and the community.

In this view, prisons and jails are not closed systems, and the attempt simply to isolate them from the external community does not seem to be an efficient strategy to limit COVID-19 spread inside the facilities, without the implementation of a testing strategy to identify and promptly isolate infected inmates and correctional workers (9–12). The application of a stochastic dynamic COVID-19 transmission model to a large US jail allowed to estimate that asymptomatic testing, together to depopulation and single celling, was an effective measure to reduce ~83% of the expected cases in inmates and correctional workers (13). In this sense, antigen-testing screening seems to be more appropriate to the specificity of correctional and detention facilities than molecular testing, because it is less expensive, provides results more rapidly (30–60 min), does not require specialized personnel to perform it and finally, allows a large number of subjects to be tested in a short period (14).

The aim of the study is to investigate the efficacy of a protocol instituted for the prevention and control of COVID-19 infection in an Italian correctional facility for inmates affected by chronic diseases. The protocol involved two screening surveys by antigen tests performed in all the prisoners and correctional workers.

## Methods

### Facility Design

The study was performed at the Bari (Apulia, Italy) correctional facility. At the time of the survey, it had a capacity of 299 male residential inmates, with an average occupancy rate of 122%. The penitentiary includes four sections and is mainly oriented toward the treatment and rehabilitation of prisoners with chronic diseases. In addition to the sections, therefore, a medical service operates in a diagnostic-clinical area, including an integrated health care service with 12 two-beds cells, ambulatories, and a room for rehabilitation and physiotherapy.

In the sections, the number of inmates per cell ranged from two to six, at least 3 square meters of floor space per inmate always being guaranteed. Although there is a canteen for the preparation of meals, there are no common areas for their consumption, and inmates eat in their cells. They are allowed to cook in their cells and to receive food packages from the outside. In all the cells there are toilets in a separate space. Some of the inmates perform different jobs within the institution, as food carriers, housekeepers, laundry attendants, and personal assistants of other disabled inmates. A single personal assistant can take care of more than one prisoner with special medical needs.

**Figure 1:**
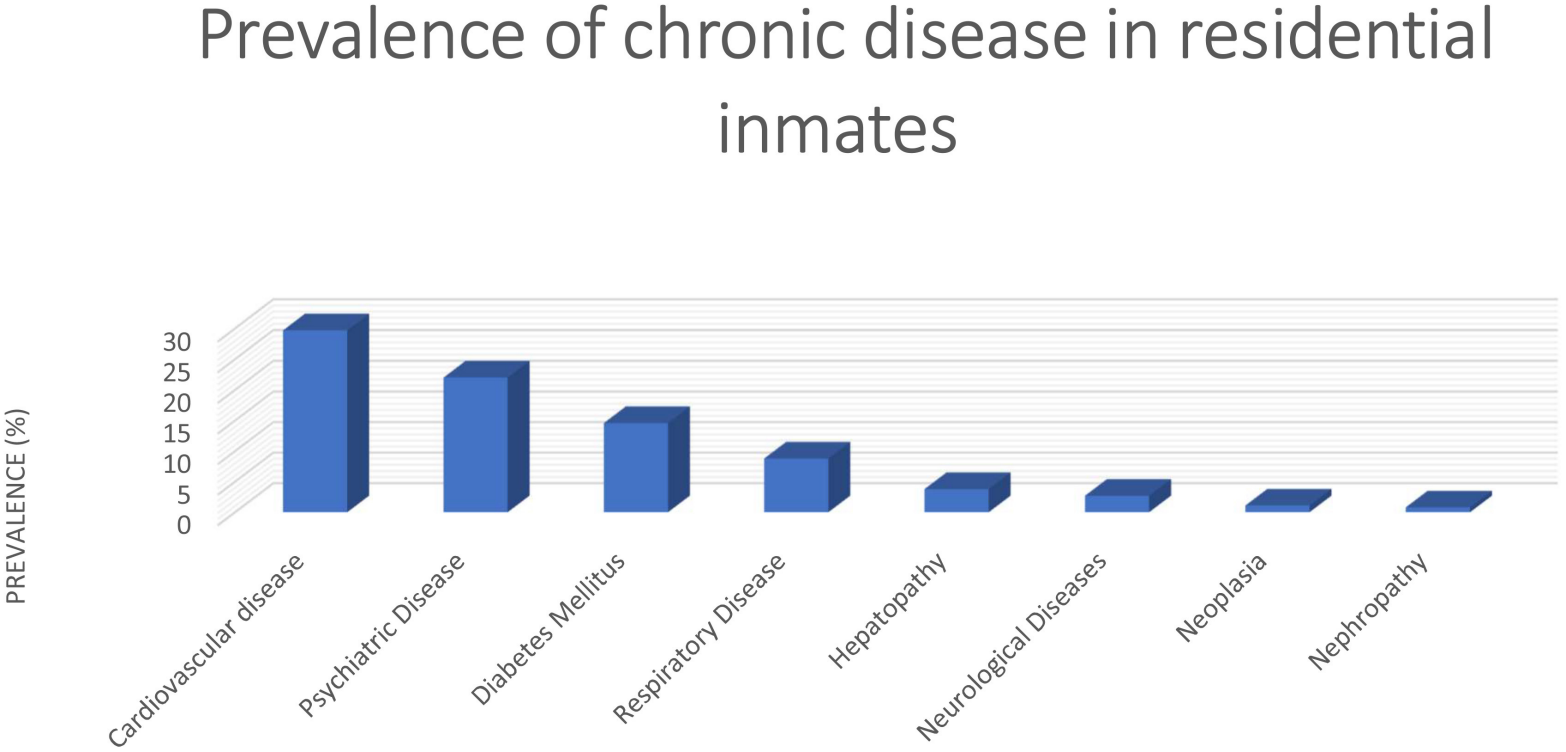
Prevalence of chronic diseases in residential inmates at the investigated correctional facility.

Most of the residential inmates have at least one chronic disease, and some of them suffer from severe disabilities (Figure 1).

Correctional workers at the investigated facility include: correctional officers (CO), administrative staff (AS), operators working with people completing their sentence outside the prison (OOP), correctional health care workers (HCW), employed exclusively in the facility, or spending a part of their working time inside the prison.

### COVID-19 Prevention Measures

**Figure 2:**
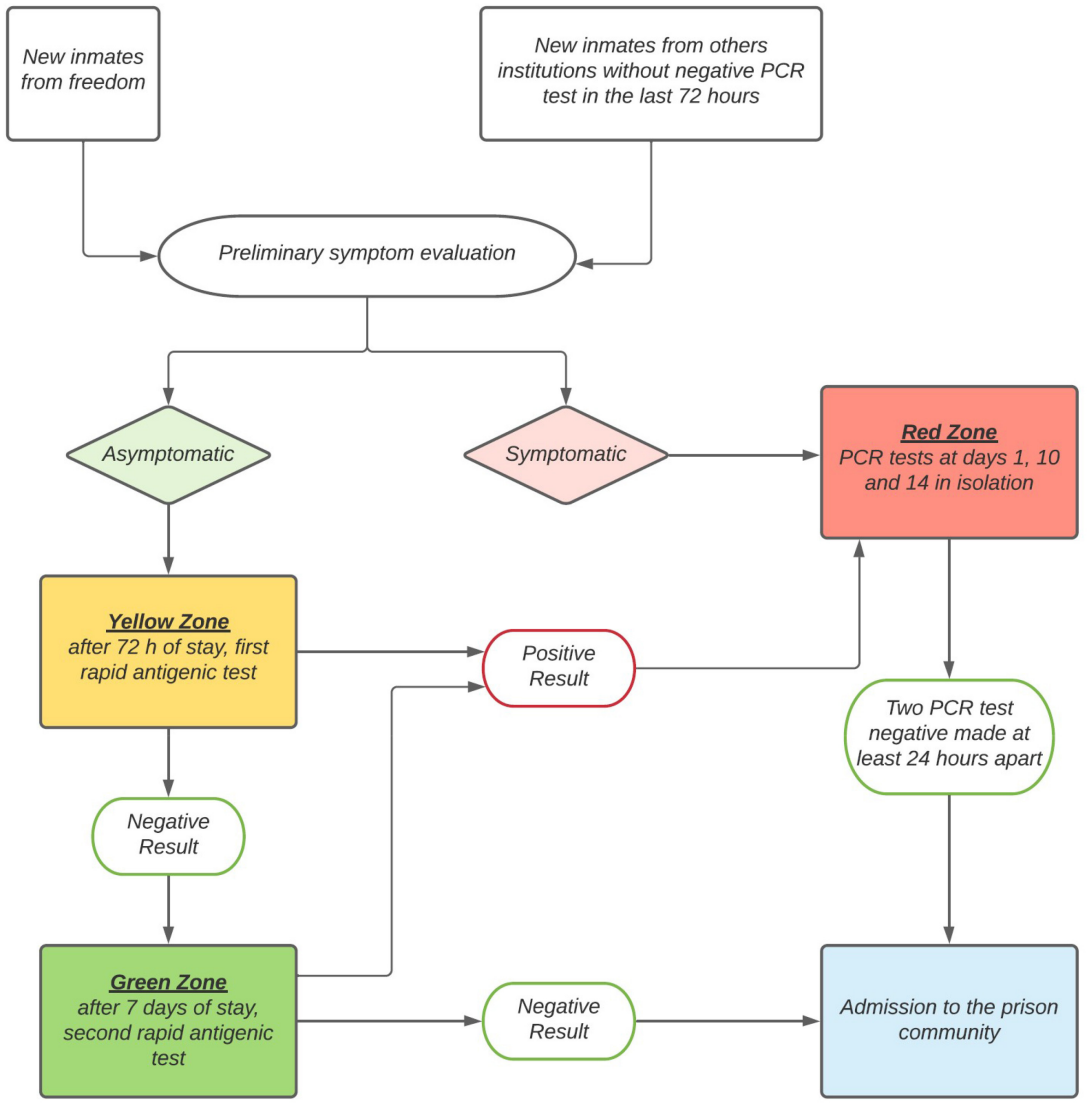
Filter areas system for the safe entry into the penitentiary for new inmates.

In order to prevent the spread of COVID-19 infection, preventive procedures were introduced in March 2020. Firstly, specific procedures were instituted to ensure safe entry into the penitentiary for new inmates coming in from the community or from other correctional institutions. In detail, protected separate pathways were created, new inmates were obliged to disinfect their hands and wear a certified medical mask prior to entry, and a new filter area system (Figure 2) was introduced, including three different filter areas:

- yellow area: dedicated to asymptomatic new inmates; in this area, they underwent the first antigen test. If negative, they were then allocated to single cells for 72 h, following which a second antigen test was performed. In cases when the first or second test was positive, the new inmates were immediately moved to the red area.

- Green area: dedicated to quarantine for 7 days of the new inmates from the yellow area who had a negative second test. At the end of the 7th day, they underwent a new antigen test and, if negative, they were admitted to the prison community.

- Red area: dedicated to the new inmates with suspect COVID-19 cases. They were kept in this area and immediately tested by rRT-PCR test, that was repeated after 10 days of isolation. If both the tests were negative, they were admitted to the prison community in the following 48 h, while in cases of a positive result on entry but negative after 10 days, they were kept in isolation and repeated the rRT-PCR test on the 14th day. However, admission to the prison community for previously rRT-PCR positive cases was permitted only after two negative rRT-PCR tests, performed at least 24 h apart. The red area was also reserved to residential inmates with suspect symptoms and to confirmed COVID-19 cases.

Since the beginning of the COVID-19 emergency, prevention measures had also been instituted inside the sections. All the internal spaces dedicated to social interchange, including the church and the areas dedicated to training and schooling, were closed, while the walkways and the outdoor common spaces remained open, separately for each section, and could be used for at least 4 h per day. Meetings with relatives or lawyers took place in the dedicated areas equipped with separator screens and physical contact with external visitors was forbidden. If a contact occurred, the detainee was isolated for 72 h, after which a rRT-PCR test was performed before readmitting him to his section.

Finally, specific prevention measures were instituted to protect correctional workers. All staff, both CO and HCW, working in the yellow, green and red areas use full personal protective equipment (PPE), including a disposable protective gown, face shield and goggles, FFP2 respirators, disposable gloves, and shoe covers. Outside these areas, all the correctional workers wear FFP2 respirators, disposable gloves and shoe covers, and all the detainees have to wear mandatory medical certified masks during matriculation procedures, meetings with visitors, video hearings, medical examinations. All correctional workers were taught basic COVID-19 knowledge and were trained in basic protective measures, hand hygiene practice and use of PPE. A health check filter and temperature measurement were implemented for all the correctional workers, external workers and visitors who entered the prison. In addition, all the correctional workers were asked to conduct self-health monitoring, and in cases of symptoms known to be suspect for COVID-19, they were asked to report them immediately, to remain in quarantine at home, and to undergo testing. In case of close contact with a COVID-19 confirmed case outside the prison, all the correctional workers were immediately quarantined and return to work was possible only 14 days since the last contact or 10 days if showing a negative rRT-PCR test, according to the Italian regulation.

### Survey Design and Study Population

Two antigen testing campaigns were carried out in the period 10 November – 09 December 2020, and 10 December 2020 – 27 January 2021, respectively, enrolling all the residential and new inmates present in the correctional facility at the time, and all the correctional workers employed at the facility, including CO, HCW, AS, and OOP. External workers and judges that attended the prison during the study period were also included in the study population. In the second campaign, every subject underwent the antigen test at least 30 days after the first test.

Participation in the study was on a voluntary basis. Each subject was administered a questionnaire by trained medical personnel, probing demographic information, work tasks, previous, or current symptoms compatible with COVID-19 infection, and previous possible exposure to confirmed or suspected COVID-19 cases.

Specimens for antigen testing were collected by flocked nasopharyngeal (NP) swab, placed in extraction tubes with sodium chloride, sodium azide as a preservative in Tris-HCL buffer. The samples were collected by trained health personnel in the sanitary area of the correctional facility. All the subjects with confirmed positivity to the antigen test underwent confirmation by rRT-PCR test on samples collected within 1 h after the positive antigen test. Specimens for rRT-PCR testing were collected by NP and oropharyngeal (OP) swab, placed together in a 3-ml tube of universal transport medium (UTM-RT System; Copan Diagnostics, Murrieta, CA, USA). Only subjects with a confirmed rRT-PCR positive test were considered as confirmed cases. All subjects positive at the first campaign were excluded from the second campaign.

### Laboratory Analysis

Antigen test analysis was performed in the medical area of the correctional facility by trained health care personnel, by fluorescence immunoassay (FIA) for the qualitative detection of specific SARS-CoV-2 nucleocapsid protein antigen. The cut-off value of the instrument is generally 1, but in this survey, tests showing a value ≥0.75 were considered positive. According to this approach, no further confirmation or quality control was applied for the subject with negative antigen tests. A sensitivity of 87.5% and a specificity of 97% were reported for the analytical method used.

rRT-PCR assay was performed in a laboratory accredited by the Local Health Authority, targeting SARS-CoV-2 *E-, RdRP-*, and *N-*gene, according to the WHO protocol (15). Briefly, NP/OP swabs were subjected to nucleic acid extraction with the MagNA Pure System (Roche Diagnostics), in accordance with the manufacturer's instructions. The presence of the SARS-CoV-2 genes was identified by a commercial rRT-PCR assay (Allplex 2019-nCoV Assay; Seegene). Amplification and detection were performed for 45 cycles on a Biorad CFX96 thermocycler (Biorad Laboratories, the Netherlands). Samples were considered positive at molecular screening if all three E-, N-, and RdRP genes were detected.

### Statistical Analysis

Statistical analysis was performed using the SPSS program (version 14, Chicago, IL, USA). For descriptive statistics, continuous variables were expressed as median and range, and non-continuous variables as frequencies and percentages. Parametric tests or non-parametric tests were used for the analysis. Statistical significance was set at *p* < 0.05.

## Results

In total, 426 inmates were eligible for the first testing campaign, including 64 new and 362 residential detainees, 23 of which were staying in the integrated health care service. Across groups, 100% of inmates agreed to participate in the first campaign. Median age was 36.7 years for the new inmates and 42.2 years for the residential inmates, and the majority of participants was Caucasian (96.9% of the new and 95.3% of the resident inmates). For the second campaign, 480 inmates were tested, 112 new and 368 residential detainees, 21 of which were hospitalized in the integrated health care service. The participation rate was 100% for the new inmates and 99.5% for the residential inmates. Median age of the new inmates was 36.8 years, and 99.1% were Caucasian, while residential inmates showed similar general characteristics to those observed in participants in the first campaign. A total of 353 detainees participated in both the campaigns, including 45 new inmates at the first campaign and 308 residential inmates.

Only two new inmates resulted positive at the first campaign and there were no positive cases at the second. Both positive cases were asymptomatic and negative at the antigen test performed at entry to the correctional facility, and then positive at the test performed 72 h after entry. Both the positive cases remained asymptomatic until recovery. During the study period, no further positive cases were observed among the inmates outside of the testing campaigns (Table 1).

**Table 1:** General characteristics of the inmates, correctional workers subdivided by job, and external workers participating in the study, and positive results to the two screening campaigns.

	**First screening campaign**				**Second screening campaign**			
	**Number positive/total**	**Age (years)^**a**^ median (range)**	**Gender^**a**^ Male (%)**	**Ethnicity caucasian (%)**	**Number positive/total**	**Age (years)^**a**^ median (range)**	**Gender^**a**^ male (%)**	**Ethnicity caucasian (%)**
Residential inmates	0/362	42.2 (19.0–82.8)	100.0	95.3	0/368	40.9 (19.1–82.9)	100.0	96.5
New inmates	2/64	36.7 (19.4–66.3)	100.0	96.9	0/112	36.8 (18.4–76.1)	100.0	99.1
Correctional officers	4/216	50.8 (23.5–61.0)	85.6	100.0	0/202	50.3 (23.6–61.2)	85.1	100.0
Correctional HCW	2/77	38.8 (23.9–61.6)	31.2	100.0	0/47	43.6 (24.2–61.2)	29.8	100.0
Administrative staff	0/66	53.2 (33.1–65.8)	74.3	100.0	0/49	52.3 (33.2–66.0)	73.5	100.0
OOP					0/23	53.8 (34.5–62.1)	43.5	100.0
External workers	0/8	54.2 (40.4–62.6)	62.5	100.0	0/4	54.3 (44.1–62.7)	50.0	100.0
Total	8/793	47.3 (19.0–82.8)	86.9	97.6	0/805	46.6 (18.4–82.9)	88.7	98.3

a *p < 0.001 HCW, Health Care Workers; OOP, Operators working with people completing their sentence outside the prison*.

A total of 367 correctional workers was tested at the first campaign, consisting of 216 CO, 77 HCW, 66 AS, and 8 external personnel who usually entered the detection facility. The participation rate was 90.0% for CO, 96.3% for HCW, 89.2% for AS, 100% for external personnel. Median age for these participants was lower for the correctional HCW (38.8 years) than for the other groups, in which median age was over 50 years. All the correctional workers were Caucasian, with a prevalence of male for CO (85.6%) and AS (74.3%), but not HCW (31.2%). At the second campaign, 325 correctional workers, consisting of 202 CO, 47 HCW, 49 AS, 23 OOP, and 4 external personnel, were tested. The participation rate was 85.6% for CO, 59.5% for HCW, 74.2% for AS, 100% for OOP and external workers. OOP were prevalently male (43.5%), with a median age of 53.8 years, while for the other groups the general characteristics were similar to those observed in the first campaign (Table 1). Age and gender, but not ethnicity, showed a significant difference among the groups (including inmates), both at the first campaign and at the second (*p* < 0.001). A total of 182 CO, 44 HCW, 47 AS, and 2 external workers participated in both the campaigns.

In the first testing campaign 4 CO and 2 HCW resulted positive, while no new positive cases were observed in the second campaign (Table 1). The frequency of positive cases was not significantly different among the groups, including inmates (*p* = 0.06). All the positive cases were asymptomatic at the testing, then one HCW referred the onset of fever, coughing, and muscle pain during the isolation period at home. During the study period, 1 CO, and 1 HCW were also recorded as positive outside the testing campaign for the onset of symptoms while at home. All the high and low risk contacts were further tested and resulted negative. Regarding the activities performed by the positive workers during the study period, 3 of the CO acted as escort guards, and 2 of the HCW performed part of their working activity outside the correction facility. None of the positive cases referred high or low risk contact with any of the other positive cases.

## Discussion

The present study showed that the implementation of a full risk management plan, including an antigen test screening program, was able to prevent COVID-19 outbreaks in a correctional facility.

The two testing campaigns reported in this study were performed in the period November 2020—January 2021. During the study period, a marked increase in the daily incidence of new COVID-19 cases was observed in Apulia, the region where the correctional facility is located, despite the implementation of more restrictive measures at National and Regional level (16). Particularly, in Apulia, the total number of COVID-19 new confirmed cases in the study period was equal to 86,457, with a cumulative incidence rate of 2196.6 per 100,000 inhabitants and a daily incidence ranging between 5.6 and 47.9 per 100,000 inhabitants (17).

However, despite the presence in the investigated prison facility of specific risk factors, such as overcrowding leading to the difficulty for respecting social distancing and the presence of particularly vulnerable inmates, none of the residential prisoners became infected, although positive COVID-19 cases were detected both in new inmates and in correctional workers. These results could be linked to the strict application of the prevention procedures implemented since the beginning of the Italian pandemic, and in particular, to the adoption of an innovative system of filter areas (Figure 2) to avoid entry into the correctional community of both mild symptomatic and pre- or asymptomatic new inmates. The observed results seem also to be in agreement with the fact that no outbreaks have been reported within the investigated prison since the beginning of the pandemic.

Our findings seem to be in contrast with the dramatic situation reported in prisons and jails worldwide. At least 3,300 inmates and 5,100 correctional workers were reported to be infected by COVID-19 across Europe, but the real numbers of infections could be much higher, as not all the administrations provided data (18). Moreover, according to the US COVID Prison project, in August 2020, 90 of the largest 100 US cluster outbreaks occurred in prisons, COVID-19 case rates being substantially higher and escalating much more rapidly in prison than in the US population (19). Mass testing by rRT-PCR in selected US penitentiaries revealed wide COVID-19 outbreaks, with infection rates exceeding also 80% (20). Correctional facilities have proved to be areas of heavy SARS-CoV-2 spread also in Latin America, where the high number of positive cases observed should be considered underestimated, given the general lack of testing capacity which makes it impossible to know the true extent of the epidemic in this context. Recent evidence supports a faster viral spread of COVID-19 in Brazilian prisons than in the general population, suggesting the important role of transmission from pre-symptomatic cases inside the prison complex (21). Some scientific reports, however, seem to agree our results, showing that the introduction of specific strategies to minimize COVID-19 transmission can achieve a low number of cases in correctional and detention facilities, even in geographic areas characterized by a wide spread of the epidemic in the general population (2, 22, 23). Our findings, therefore, seems to implicate that COVID-19 management within correctional and detention facilities is possible, leaving the incompatibility with the prison regime only to a few specific cases of inmates.

One of the main strength of our study is that it included all the correctional workers, considering that COVID-19 in correctional facilities should be considered as a unique occupational and public health concern (24). In fact, the highest number of positive cases, even if extremely limited, was found among CO (1.9%) and HCW (2.6%). The main challenge in terms of COVID-19 prevention strategies in correctional facilities, therefore, seems to be protecting the correctional workers, that should be the main potential carriers of SARS-CoV-2 infection within the facilities (25, 26). A possible critical point in the prevention measures is the activity of escorting inmates, considering that 3 of the positive CO performed this task, that cannot always be accomplished in full conformity with physical distancing (11). Regarding the HCW, activities performed in other health care settings than the correctional facility could be possible sources of contagion. The impact of this situation should be evaluated in the organization of prison health care services during the pandemic (27).

Although rRT-PCR continues to be the gold standard for the diagnosis of COVID-19, the complexity of the method may be a limitation when the epidemiological situation requires an increase in testing capacity, particularly in congregate settings where the speed of transmission could be considerably higher and rapid contact tracing of positive cases is a major goal. The use of antigen tests, instead, allows quick evidence of infection (15–30 min), at lower cost and without the need for skilled personnel, even if it does not have the same sensitivity as rRT-PCR test, and can give false negative results (28, 29). This happens particularly if the antigen test is used outside the indications for which it is approved, namely the diagnosis of confirmed cases from 2 days before, up to 5–7 days after the onset of symptoms. The antigen test used in this study reported a high sensitivity (87.5%), whereas a previous survey showed sensitivity of 41.2% among asymptomatic subjects tested by FIA (30). The main limitation of the study, therefore, is related to the possibility that not all the asymptomatic cases may have been identified by the antigen testing campaigns. However, the results of the antigen test should be interpreted according to the epidemiological situation of the study population, because in a high-prevalence setting they have a high positive predictive value (14). Therefore, since at the time of the investigation the Apulia region was burdened by a high incidence, and since by definition the prison is a congregate setting with a presumed high incidence in the case of outbreaks, the antigen test in this context still resulted an adequate tool for the detection of COVID-19 cases.

Previous studies have described the usefulness of rRT-PCR mass testing in US or UK correctional facilities to mitigate and manage COVID-19 outbreaks by the identification of presymptomatic or asymptomatic cases (1, 20, 31–33). However, to the best of our knowledge, our study showed the first experience of a serial mass campaign by antigen tests performed both in inmates and correctional workers. Marco et al. has previously described an antigen screening test applied only to the inmates of a Spanish correctional facility, after the diagnosis of three symptomatic COVID-19 cases within a section. Antigen tests were able to find only 9 new positive cases on 81 asymptomatic inmates, while rRT-PCR performed 3 days later was able to identify 27 further asymptomatic confirmed cases (34). Apart from false-negative antigen tests, the different time of the two tests may also explain these results.

In our experience, however, the lack of new confirmed cases at the second testing campaign seems to indicate that the rapid identification and isolation of the confirmed cases in correctional workers and new inmates during the first campaign helped to avoid possible outbreaks or further new cases. In fact, our findings highlight the utility of serial cohort-based approaches to COVID-19 screening in the congregate settings, also for antigen test. Early identification and isolation of SARS-CoV-2 infection may reduce the likelihood of transmission, identifying asymptomatic and pre-symptomatic subjects that would be missed by symptoms screening alone, also supporting the medico-legal evidence that measures have been taken to avoid the onset of an outbreak in the facility (28).

The findings of our study show some further limitations for the evaluation of the efficacy of the measures implemented in the investigated prison. Firstly, our investigation relied on antigen testing and confirmatory molecular rRT-PCR analysis, which cannot identify the total disease prevalence in the correctional facility due to previous unknown infection. Moreover, each of the two testing campaigns lasted about 30 days, and although each subject was screened at least 30 days after the first investigation, there is still the possibility that some asymptomatic or mildly symptomatic subjects became infected during the time between the two testing campaigns and so were not then detected because they were already in the resolution phase of the infection.

## Conclusion

The study showed that the simultaneous application of both a strict protocol for COVID-19 prevention, including physical distancing when possible, the use of appropriate PPE for both correctional workers and inmates, timely and effective measures to isolate infected detained persons and workers, and testing campaigns, may prevent SARS-CoV-2 transmission in correctional facilities avoiding outbreaks also in the communities where correctional workers live and detained persons return when released. Particularly, serial antigen screening tests, despite the limitations with respect to rRT-PCR, might give a contribution to the containment of the COVID-19. These findings may have implications also for the evaluation to the real need of an accelerated release of inmates during the pandemic scenario.

Prison COVID-19 containment should be considered as an essential support to the overall public health response to the pandemic, also to mitigate its impact on public healthcare systems. The protection of the prison population and correctional workers could be a first step toward an enhanced protection especially of the most disadvantaged communities.

## Data Availability Statement

The original contributions presented in the study are included in the article, further inquiries can be directed to the corresponding author.

## Ethics Statement

The studies involving human participants were reviewed and approved by Ethical Committee of the Bari University Hospital (protocol n. 0099199). The participants provided their written informed consent to participate in this study.

## Author Contributions

AS, NB, and PL conceptualized and designed the study and performed the interpretation of the results. NA, DP, and FC performed the acquisition of the data. FC and LD performed data analysis. AS and PL wrote the manuscript. AS, FC, and PL prepared the figures. All authors have read and approved the final manuscript.

## Conflict of Interest

The authors declare that the research was conducted in the absence of any commercial or financial relationships that could be construed as a potential conflict of interest.
